# Editorial: Role of Inflammation in Neurodegenerative Diseases

**DOI:** 10.3389/fimmu.2022.958487

**Published:** 2022-06-21

**Authors:** Maya Koronyo-Hamaoui, Bhakta Prasad Gaire, Sally Ann Frautschy, Jorge Ivan Alvarez

**Affiliations:** ^1^ Department of Neurosurgery, Maxine Dunitz Neurosurgical Research Institute, Cedars-Sinai Medical Center, Los Angeles, CA, United States; ^2^ Department of Biomedical Sciences, Division of Applied Cell Biology and Physiology, Cedars-Sinai Medical Center, Los Angeles, CA, United States; ^3^ Geriatric Research Education and Clinical Center, Veterans Greater Los Angeles HealthCare System, Los Angeles, CA, United States; ^4^ Department of Neurology, University of California, Los Angeles, Los Angeles, CA, United States; ^5^ Department of Medicine, University of California, Los Angeles, Los Angeles, CA, United States; ^6^ Department of Pathobiology, School of Veterinary Medicine, University of Pennsylvania, Philadelphia, PA, United States

**Keywords:** neuroimmunology, Alzheimer’s disease, multiple sclerosis, neurodegeneration, neuroinflammation, autoimmune disease, neurobehavioral deficits, biomarkers

A precise and dynamic immune response is pivotal for maintaining defense against pathogens, cellular homeostasis, and tissue repair following injury. Dysregulated immune responses, on the other hand, can cause chronic inflammatory and/or autoimmune diseases, and contribute to degenerative pathologies and even organ failure. Neuroinflammation during diverse central nervous system (CNS) diseases is mainly governed by activation of CNS resident glial cells (i.e. microglia and astrocytes) and infiltrated peripheral immune cells (macrophage, neutrophils, natural killer cells, and lymphocytes), through a compromised blood-brain barrier (BBB), blood-meningeal barrier or *via* other routes (choroid plexus and circumventricular organs). Upon activation, these cells are involved in the production of inflammatory mediators such as cytokines, chemokines, matrix metalloproteinases, among others, which requires tight regulation to minimize tissue damage associated with chronic activation. In the past two decades, emerging evidence has driven a paradigm shift in our understanding of CNS inflammation, revealing novel neuroprotective roles of alternatively activated microglia, astrocytes and infiltrating immune cells in neurological disorders (1–14).

In this special Research Topic, we called for studies, which would reflect roles of inflammation in neurodegenerative diseases. We received a total of 32 articles comprising 6 reviews, 2 mini-reviews, 22 original research articles, and 2 case reports. Among them, 27 articles were published in *Frontiers of Immunology*, whereas 5 were published in *Frontiers in Neurology*. These articles cover some of the exciting findings highlighting the critical and diverse roles of inflammation in neurodegenerative diseases, mainly in Alzheimer’s disease (AD) and multiple sclerosis (MS).

In the context of AD, a common view has been that neuroinflammatory responses derive pathogenic processes affecting disease onset and progression. Based on this notion, attenuating neuroinflammation would be primarily beneficial in preventing or halting AD (15, 16). Indeed, Hickman et al. reported the neuroprotective potential of microglial C-X3-C motif chemokine receptor 1 (CX3CR1) depletion in the double-transgenic mouse models of AD carrying human AD mutations in presenilin 1 and amyloid precursor protein (PS1-APP). This study found that CX3CR1 deficiency in microglia is associated with reduced plaque load, amyloid β-protein (Aβ) levels, and neurobehavioral deficits. Similarly, Barron et al. showed the harmful effects of systemic inflammation by lipopolysaccharides (LPS) injection in neurobehavioral functions employing a mouse model of tauopathy. Another report by Joly-Amado and collages. also using a mouse model of tauopathy, revealed that overexpression of chemokine C-C motif ligand 2 in the brain triggered neuroinflammatory glial activation and accelerated tauopathy.

Other studies demonstrated the neuroprotective effects of enhancing or modulating the immune responses to fight AD. Ma et al. uncovered a neuromodulatory role of a novel fatty acid that may have particular efficacy for AD associated with the most common genetic risk factor apolipoprotein E4 (APOE4). The authors described neuroprotective effects of a diet consisting of high n-6 fatty acid linoleic acid to n-3 fatty acid ratios, a diet traditionally considered to be ‘pro-inflammatory’. However, the brains of E4FAD mice fed with this diet showed a surprising resolution of neuroinflammation. Further studies showed that the effect was mediated by its metabolite, n-6 docosapentaenoic acid, a fatty acid, typically upregulated in n-3 insufficient diets, which exerted a pharmacogenomic role that could counteract detrimental neuroinflammatory and neurodegenerative responses in APOE4-induced AD-model mice, enhancing clearance of plaques and resolution of inflammation.

In an elegant study, Li et al. described for the first time that bone marrow-derived macrophages, and moreover, glatiramer acetate-activated macrophages efficiently clear Aβ_42_ oligomers and fibrils and rescue synapses following Aβ_42_ exposure in primary cortical neurons *in vitro* and in the hippocampi of APP_SWE_/PS1_ΔE9_ transgenic murine models of AD *in vivo*. These findings provide the rationale for harnessing macrophages to treat AD. Further, Li et al. revealed that cortical neurons were far more susceptible to well-defined and stabilized Aβ_42_ oligomers than the fibrils, triggering neuritic arborization retraction, neuronal function alterations, and loss of pre- and post-synaptic markers. This study suggested that alternatively activated macrophages confer a potential synaptoprotective phenotype through augmented capacity to remove highly toxic Aβ_42_ oligomers and induce synaptic preservation and regeneration.

Several other groups demonstrated the importance of targeting microglial or peripheral myeloid cell molecules to attenuate AD pathology. Estfanous et al. revealed that elevated expression of microglial microRNA (miR)-17 hampers the autophagy-mediated Aβ degradation in AD patients. This study also reaffirmed these results in 5xFAD mice model, where they found that inhibiting miR-17 expression in microglia improved Aβ degradation and autophagy. Abdullah et al. reported the effects of nilvadipine, calcium channel blocker, against AD-influenced cognitive decline and changes in cerebrospinal fluid (CSF) biomarkers in AD patients. After adjusting for confounding effects of ApoE genotype, age and gender, nilvadapine reduced CSF Aβ_42_/Aβ_40_ ratios, phosphorylated tau P181, as well as YKL-40 and neurogranin, indices of synaptic degeneration and neuroinflammation. Another clinical study by Aliseychik et al. identified AD-specific clonotype features of T-cell receptor γ genes derived from blood and brain cells of AD patients. A report by Zuroff et al. uncovered novel effects of interleukin-34 (IL-34) on macrophage activation and phenotype. This *in vitro* study revealed that IL-34 activatation of macrophages reduced their Aβ uptake ability. IL-34 also appeared to impair monocyte differentiation into macrophages and reduce their ability to uptake pathological forms of Aβ, suggesting that IL-34 promotes Aβ accumulation and can act as pathogenic mediator in diverse neuroinflammatory diseases associated with amyloid pathology. Moreover, Ma et al. reported the importance of arginase 1 in amyloidosis. They revealed that arginase 1 deletion in myeloid cells triggers Aβ deposition, microgliosis, and behavioral deficits in mouse model of spontaneous amyloidosis.  Ma et al. .also reported the importance of myeloid arginase 1 in alleviating AD pathogenesis. They found that arginase-1 deficiency in myeloid cells up-regulates glial gene transcripts and reduces microglial ability for Aβ phagocytosis.

The above studies emphasized that manipulating macrophages to correct aberrant inflammation, and modulating neuroinflammatory responses can be viable options to counteract pathogenesis. A case report study by Nelson et al. demonstrated the importance of maintaining a delicate balance, by showing a detrimental effect of the combination of immunosuppressive drugs (prednisone and tacrolimus, a calcineurin inhibitor) following orthopedic heart transplantation in a 57-year-old man, was associated with cerebral amyloid angiopathy (CAA)-related inflammation.

Comprehensive reviews on neuroinflammation in AD and cerebral small vessel disease (CSVD) were also included in this special issue. Hampel et al. provided a broad review of the cellular and molecular mechanisms, together with the critical pathogenic aspects of neuroinflammation in AD. This review described the involved immune cell types, potential reasons for the failure of previous anti-inflammatory therapies, and the value of precision medicine in targeting neuroinflammation as a prospective AD drug discovery. Further, Kloske and Wilcock discussed the risks associated with APOE-isotype dependent neuroinflammation in AD. This review highlighted that APOE4 contributes to increased proinflammatory responses of glial cells, impaired BBB functions, triggering neurodegeneration, elevated Aβ aggregation, and increased tau hyperphosphorylation. Hence, given its damaging role in AD, targeting APOE is an exciting strategy for AD drug development. Finally, Zhou et al. reviewed the imaging aspects of neuroinflammation in AD with a focus on positron emission tomography, by which improved imaging probes for microglial or astrocytes activation (pro- or anti-inflammatory phenotypes) with highly specific ligand affinities enable *in vivo* brain neuroinflammation imaging. Further, a review provided by Jian et al. highlighted the role of immunosenescence in CSVD, in which the aging immune system plays a role in the development of CSVD.

The pathogenic aspects of neuroinflammation in MS and other autoimmune diseases were also discussed in this special Research Topic. DiSano et al. demonstrated that activated B cell-related chemokines and trophic factors were observed during chronic, progressive Theiler’s virus mouse model of MS. Importantly, Sánchez-Fernández et al. suggested that OLT1177, a selective inhibitor of the NOD-, LRR- and pyrin domain-containing protein 3 inflammasome, can attenuate symptoms of experimental autoimmune encephalomyelitis (EAE), an animal model of MS. These effects were attributed to reducing the expression levels of proinflammatory cytokines such as IL-1β, IL-18, IL-6, and TNF-α in the spinal cord of EAE mice. In addition to OLT1177-enriched diet, the prophylactic oral administration of OLT1177 significantly decreased the infiltration of CD4 T cells and macrophages in the spinal cord, suggesting the substantial anti-inflammatory properties of OLT1177. Notably, Doroshenko et al. revealed the protective role of microglial peroxisome proliferator-activated receptor-δ (PPAR-δ) in EAE mouse model employing loss-of-function approach. This study uncovered that deleting PPAR-δ in microglia exacerbates axonal injury, oxidative stress, neuroinflammation, and neurodegeneration. A clinical report by Arslan et al. showed that markers associated with thiol homeostasis as a novel oxidative stress parameter are elevated in the serum and CSF of patients with inflammatory MS and neuromyelitis optica spectrum disorders. These markers, therefore, can possibly be used for monitoring disease severity. Ayzenberg et al. studied the role of T-cell during brainstem encephalitis; and revealed that gauging T cells can be an important differential diagnosis in myasthenia gravis. Additional review manuscripts on this topic were included: Guerrero and Sicotte.provided an overview of the detrimental and neuroprotective aspects of microglia in MS, suggesting that microglia can be both friend and foe during MS pathogenesis. The authors also suggested that microglia mainly promote proinflammatory responses during the early lesion stages of MS, whereas microglia exhibit anti-inflammatory properties during late lesion stages. Similarly, Deerhake et al. reviewed both the pathogenic and protective roles of pattern recognition receptors in animal models of MS, and Ifergan and Miller highlighted the importance of peripheral myeloid cells in MS pathogenesis, suggesting that these cells are potential therapeutic targets for MS and other autoimmune disorders.

In addition to AD and MS, aspects of neuroinflammation in other neurological conditions were included in this special Research Topic. Jhun et al. showed that CD103 deficiency induced behavioral patterns, simlar to autism and attention-deficit hyperactivity disorder (ADHD) and attenuated age-related cognitive decline in murine models. CD103, an integrin and E-cadherin receptor, is predominantly expressed by CD8 T cells in the brain, gut, and other tissues. Absence of CD103 was shown to damage gut immune responses, especially the local T cell function. Next the authors found that CD103 deletion exacerbated cognitive and behavioral deficits in animal models of autism and ADHD. In another study, the neuroprotective effects of resveratrol were reported by Zheng et al. using levodopa-induced dyskinesia rat models. These neuroprotective effects were mediated by downregulating the pro-inflammatory responses of activated microglia and astrocytes. Accordingly, Ghafouri-Fard et al. studied the inflammatory factor NF-κB-related long non-coding RNA (lncRNA) in the blood of patients with Parkinson's disease (PD). This study revealed that NF-κB-related lncRNAs are associated with modulation of immune responses and apoptotic pathways. Jiang et al. reported that intranasal administration of MMI-0100, a cell-penetrating peptide inhibitor of MAPK-activated protein kinase II, attenuates neuroinflammatory responses induced by Aβ_1-42_ or LPS in the hippocampus of male Kunming mice. MMI-0100 administration attenuated LPS-induced glial activation, and dramatically decreased the expression of series of pro-inflammatory mediators such as TNF-α, IL-6, IL-1β, COX-2, and iNOS. In healthy mouse brains, Moral et al. investigated age-dependent and sex-specific functional states of microglia using multicolor two-photon imaging techniques. This study revealed the different functional states of microglia among different age groups, and suggested that caloric restriction could be a potent, cost-effective, and clinically relevant tool for rejuvenating microglia. Further, Goddery et al. revealed the important role of microglia and macrophages as antigen presenting cells that promote the infiltration of CD8 T cells when the CNS is infected with the Theiler’s murine encephalomyelitis virus. An exciting study by Kim et al., reported that gut microbiota, which consist of both pro- and anti-inflammatory bacteria, are bidirectionally connected to the brain. Indeed, the overgrowth of inflammatory bacteria such as *Escherichia coli* in the gastrointestinal tract caused neuropsychiatric disorders. While notably, a balanced gut microbiota with prominent anti-inflammatory bacteria such as *Lactobacillus mucosae* alleviated psychiatric disorders by decreasing pro-inflammatory and increasing anti-inflammatory responses.

Finally, Passaro et al. provided a systematic review on the emerging role of central and peripheral immune crosstalk in diverse neurological disorders, including stroke, traumatic brain injury, MS and other autoimmune disorders, amyotrophic lateral sclerosis, AD, PD, and glioblastoma. Taken together, this special Research Topic highlights an overview of recent findings and new insights into both the detrimental and beneficial roles of inflammation in neurodegenerative diseases. Thus, this novel understanding on how to modulate neuroinflammation could be a promising therapeutic strategy for neurodegenerative diseases.

## Author Contributions

MK-H and BPG drafted the manuscript; All authors edited and approved the manuscript for publication.

## Funding

This study was supported by NIH/NIA R01AG056478, R01AG055865 (MK-H), VA Merit BX005276 (SAF) and R01AG 066212 (SAF).

## Conflict of Interest

The authors declare that the research was conducted in the absence of any commercial or financial relationships that could be construed as a potential conflict of interest.

## Publisher’s Note

All claims expressed in this article are solely those of the authors and do not necessarily represent those of their affiliated organizations, or those of the publisher, the editors and the reviewers. Any product that may be evaluated in this article, or claim that may be made by its manufacturer, is not guaranteed or endorsed by the publisher.
